# CCAS: One-stop and comprehensive annotation system for individual cancer genome at multi-omics level

**DOI:** 10.3389/fgene.2022.956781

**Published:** 2022-08-11

**Authors:** Xinchang Zheng, Wenting Zong, Zhaohua Li, Yingke Ma, Yanling Sun, Zhuang Xiong, Song Wu, Fei Yang, Wei Zhao, Congfan Bu, Zhenglin Du, Jingfa Xiao, Yiming Bao

**Affiliations:** ^1^ National Genomics Data Center, Beijing Institute of Genomics, Chinese Academy of Sciences/China National Center for Bioinformation, Beijing, China; ^2^ CAS Key Laboratory of Genome Sciences and Information, Beijing Institute of Genomics, Chinese Academy of Sciences/China National Center for Bioinformation, Beijing, China; ^3^ University of Chinese Academy of Sciences, Beijing, China

**Keywords:** comprehensive annotation, multi-omics, individual cancer patient, databases integration, web server

## Abstract

Due to the explosion of cancer genome data and the urgent needs for cancer treatment, it is becoming increasingly important and necessary to easily and timely analyze and annotate cancer genomes. However, tumor heterogeneity is recognized as a serious barrier to annotate cancer genomes at the individual patient level. In addition, the interpretation and analysis of cancer multi-omics data rely heavily on existing database resources that are often located in different data centers or research institutions, which poses a huge challenge for data parsing. Here we present CCAS (Cancer genome Consensus Annotation System, https://ngdc.cncb.ac.cn/ccas/#/home), a one-stop and comprehensive annotation system for the individual patient at multi-omics level. CCAS integrates 20 widely recognized resources in the field to support data annotation of 10 categories of cancers covering 395 subtypes. Data from each resource are manually curated and standardized by using ontology frameworks. CCAS accepts data on single nucleotide variant/insertion or deletion, expression, copy number variation, and methylation level as input files to build a consensus annotation. Outputs are arranged in the forms of tables or figures and can be searched, sorted, and downloaded. Expanded panels with additional information are used for conciseness, and most figures are interactive to show additional information. Moreover, CCAS offers multidimensional annotation information, including mutation signature pattern, gene set enrichment analysis, pathways and clinical trial related information. These are helpful for intuitively understanding the molecular mechanisms of tumors and discovering key functional genes.

## 1 Introduction

Cancer is one of the leading causes of human death all over the world (Jemal et al., 2007; Ferlay et al., 2013; Torre et al., 2016). The occurrence and development of each cancer is driven by a unique set of abnormalities in its genome (Stratton et al., 2009; Garraway and Lander, 2013; Birkbak and McGranahan, 2020). Therefore, dissecting changes in the cancer genome at the multi-omics level could significantly improve our understanding of the molecular mechanisms of tumorigenesis and help the development of new treatments (Tebani et al., 2016; Olivier et al., 2019). To date, a series of large cancer genome sequencing projects have been launched as the next generation sequencing (NGS) technology becomes more and more widely used in cancer researches (Cerami et al., 2012; Gao et al., 2013; ITP-CAoWG, Consortium, 2020). Genome annotation, as an effective approach, provides a comprehensive perspective of cancers’ abnormalities by using multi-omics data. However, there are still a number of challenges that need to be addressed. Firstly, inter-tumor heterogeneity is increasingly recognized as a serious barrier in annotating cancer genome at the individual patient level. Secondly, comprehensive annotation relies heavily on existing data resources that are often located in different data centers or research institutions, which poses a huge challenge to integrate those resources. Finally, additional essential knowledge such as clinical trials, drug interactions, literature of the abnormalities are needed because they have far-reaching significance for understanding tumors.

In order to make cancer genome annotation convenient and efficient, several tools, online databases, and web servers have been developed over the past decades. ANNOVAR (Wang et al., 2010), Ensembl-VEP (McLaren et al., 2016), and SnpEff (Cingolani et al., 2012) were developed as annotation tools for variants function based on population frequencies in normal or disease cohorts, as well as damage predictions at genomic level. PCAWG-Scout (Goldman et al., 2020a), UCSC Xena (Goldman et al., 2020b), and OpenCRAVAT (Pagel et al., 2020) were designed for complex visualization and analysis services of large scale cancer datasets. PCGR (Nakken et al., 2018), GenomeChronicler (Guerra-Assuncao et al., 2020), and PORI (Reisle et al., 2022) were developed for cancer genome annotation at the individual patient level, providing many useful functions, such as mutation signature analysis, mutation burden analysis, drug interactions, as well as clinical trials analysis. However, these tools are more focused on parsing genomic level data, while lacking comprehensive annotations based on the integration of multiple cancer-related databases, or have limitations in data analysis at the individual patient level.

Here, we present Cancer genome Consensus Annotation System (CCAS), which is a comprehensive annotation server for individual cancer genome at multi-omics level. CCAS builds two ontology frameworks and integrates 20 data resources, which are commonly used in cancer researches. Information and knowledge in CCAS can be classified into 6 aspects: genomics, disease, normal/cancer cohorts, clinical trials, literature, and drug interactions (Supplementary Table S1), enabling comprehensive annotation at the individual patient level. The integration of these information allows CCAS to annotate not well studied abnormalities in patient-specific cancer subtypes by transferring knowledge across cancer subtypes and databases. Moreover, CCAS uses a two-step process to identify key functional genes that significantly change in the individual patient and play important roles in tumorigenesis. Furthermore, CCAS offers analysis including mutation signature pattern, gene set enrichment analysis. Overall, CCAS is aimed at annotating cancer genome precisely and effectively in the individual patient level.

## 2 Materials and methods

### 2.1 Data collection

**FIGURE 1:**
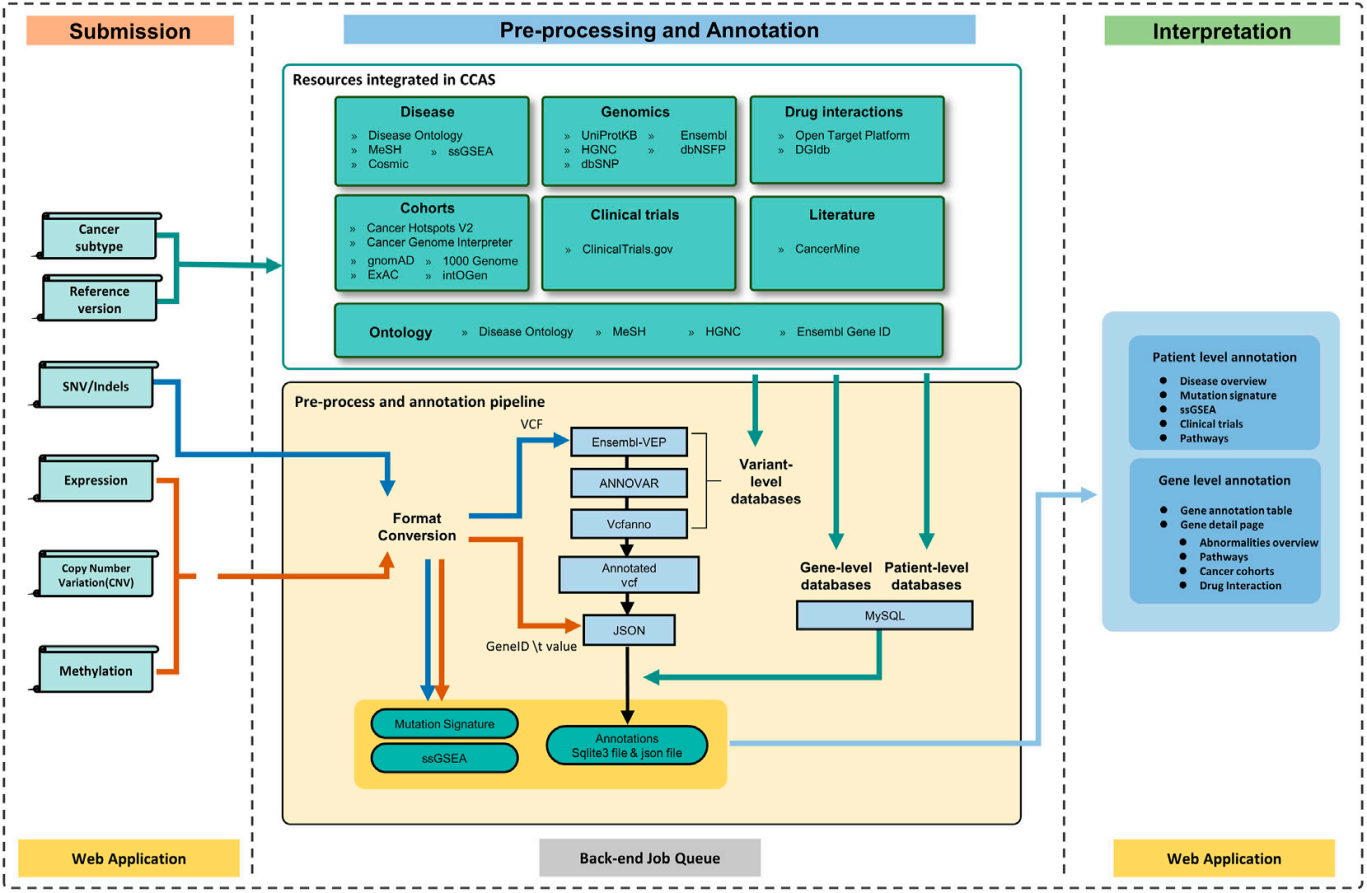
The workflow of CCAS can be divided into three modules: Submission, Pre-processing and Annotation, and Interpretation. After the user submits data to CCAS, CCAS first converts the format of the files. SNV/Indels data will be converted to VCF format and other data types will be converted to “Gene ID \t Value” format. CCAS will then annotate the patient data using the integrated data sources at multiple levels. Mutation Signature and ssGSEA calculations are also performed. The annotation results are stored in sqlite3 database (a single file database) and json file. CCAS has built user-friendly interface to help users navigate and interpret the annotation results, enabling efficient identification of key functional genes at the individual patient level.

To provide high-quality annotation results, CCAS integrated 20 resources (Supplementary Table S1) to build the annotation results at 6 aspects including genomics, disease, normal/cancer cohorts, clinical trials, literature, and drug interactions (Figure 1). Genomics aspect data were collected from Ensembl (Zerbino et al., 2018), dbNSFP (Liu et al., 2020), dbSNP (Sherry et al., 2001), HGNC (Tweedie et al., 2021), and UniProtKB (UniProt, 2021). Those resources provided knowledge of gene descriptions, IDs (gene IDs, protein IDs, and variant IDs) in different databases, protein function descriptions, and protein damage predictions. For the disease aspect, data were integrated from COSMIC (Tate et al., 2019), Disease Ontology (Schriml et al., 2022), MeSH (Baumann, 2016), single sample GSEA (ssGSEA) (Subramanian et al., 2005) and Reactome (Gillespie et al., 2022). Those data provided insights of patient’s cancer subtype including disease description, disease synonymous names, disease ontology name, and related pathways. Besides, the mutation signature analysis and ssGSEA analysis were used to reveal underlying biological processes of the patient. For the normal/cancer cohorts aspect, ExAC (Karczewski et al., 2017), gnomAD (Karczewski et al., 2020), intOGen (Martinez-Jimenez et al., 2020), the 1000 Genomes Project (Genomes Project et al., 2010), Cancer Hotspots V2 (Chang et al., 2016; Chang et al., 2018), Cancer Genome Interpreter (Tamborero et al., 2018) were collected. Those data provided the frequencies of variants both in cancer and normal cohorts. The ClinicalTrials.gov (https://clinicaltrials.gov/ct2/home) database was integrated into Clinical trial aspect. Those data provided related clinical trials information of patient’s cancer subtype including study design, eligibility criteria, and intervention. The Literature aspect was built mainly in the aid of the CancerMine (Lever et al., 2019) database, providing relationships between genes and cancer subtypes. DGIdb (Freshour et al., 2021) and Open Target Platform (Koscielny et al., 2017; Carvalho-Silva et al., 2019) were used to build the Drug interactions aspect, providing potential drug interactions of abnormalities. Disease Ontology (Schriml et al., 2022), MeSH (Baumann, 2016), Ensembl (Zerbino et al., 2018), and HGNC (Tweedie et al., 2021) databases were used to build the ontology frameworks which were used to integrate data from multiple resources. The detailed description of the databases can be found at CCAS documentation (https://ngdc.cncb.ac.cn/ccas/docs/#/, 2.3 Data sources integrated into CCAS).

### 2.2 Ontology frameworks construction

To integrate multiple data sources, we built two ontology frameworks respectively: ontology of cancers and genes. For the ontology of cancers, we downloaded data from Disease Ontology and parsed them by the Pronto package (https://pypi.org/project/pronto/). Cancer subtypes with MeSH IDs were recursively extracted starting from the node “cancer” (DOID: 162). Ultimately, 395 cancer subtypes were integrated into the CCAS. To make it easier for users to specify cancer types, all cancer subtypes were manually classified into 10 groups according to the human tissue type. MeSH terms corresponding to each cancer were fetched by using NCBI’s E-utilities. For ontology of genes, we retrieved gene IDs from the Ensembl database (release version 104) and converted them to NCBI gene IDs and UCSC gene IDs by using the HGNC database.

### 2.3 Data standardization and integration

Human protein records were extracted from UniProtKB’s XML file using Python library of BeautifulSoup4. Data in Open Target Platform, DGIdb, CancerMine, and intOGen were downloaded in tabular format. Pathway information along with diagrams were extracted from the Reactome database. Data from Cancer Hotspots V2 were converted into the VCF format and indexed by Tabix (Li, 2011) after sorting by chromosomes. For the ClinicalTrials.gov database, NCT ID (Clinical trial ID) and other metadata were extracted by the Python XML module. After that, clinical trials with drugs were retained. The MeSH terms in clinical trial records were linked to Disease Ontology by MeSH IDs. Data in Ensembl, dbNSFP, 1000 Genomes Project, ExAC, gnomAD, and dbSNP were retrieved by using Ensembl-VEP (McLaren et al., 2016) and ANNOVAR (Wang et al., 2010). CrossMap tool was used to convert data with different genome coordinates (Zhao et al., 2014).

### 2.4 Overall workflow of CCAS

The overall workflow of CCAS can be divided into three modules: Submission, Pre-processing and Annotation, and Interpretation (Figure 1).

**FIGURE 2:**
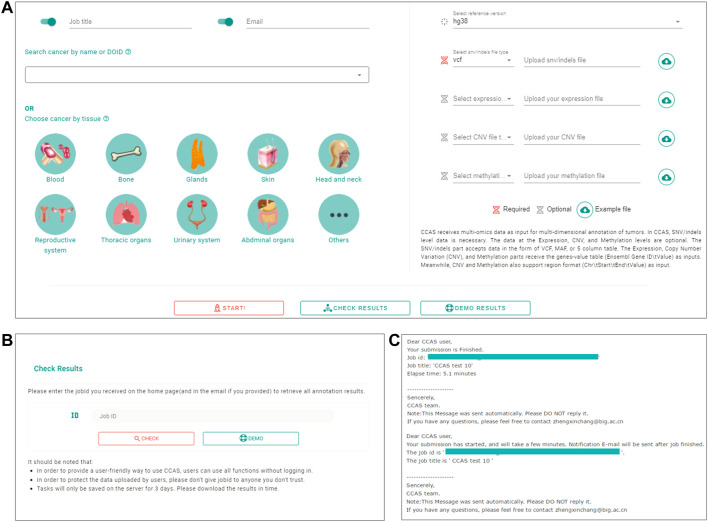
The workflow of submitting data and checking job progress in CCAS. **(A)** Submitting portal at home page. CCAS receives data at multi-omics level including SNV/Indels (required), Expression, Copy Number Variation (CNV), and Methylation along with, job title, notification email, the Disease Ontology ID, and reference version. **(B)** Check results page in CCAS. On this page the user can check the progress of the job. **(C)** Notification emails sent to users at the start of a job and at the end of a job.

The Submission module was used to collect user’s uploaded data as well as the reference genome version and cancer subtype of the patient (Figure 1). A submission portal was built to provide user-friendly interface at the home page of the web application. Cancer subtype can be selected at the left part of portal. The multi-omics data files along with reference genome version can be uploaded at the right part of the submission portal (Figure 2A). The mandatory inputs were reference genome version, cancer subtype, and the data file in SNV/Indels level.

The Pre-processing and Annotation module consists of four main parts: format conversion, variant level annotation, gene level annotation, and patient level annotation (Figure 1). At the format conversion part, SNV/Indels level files including mutation annotation format (MAF) or 5 columns tabular (5coltsv) format were converted into the VCF format. “chr” prefix was added if it did not exist. For data in expression, CNV and methylation level, files in region format were converted to 2 columns table (“Ensembl Gene ID \t Value”) by using bedtools (https://bedtools.readthedocs.io/) (Figure 1). At the variant level annotation part, data in SNV/Indels level were annotated. the pipeline integrated ANNOVAR, Ensembl-VEP, Vcfanno (Pedersen et al., 2016), vt-normalize (Tan et al., 2015), DeconstructSigs (Rosenthal et al., 2016), GSVA package (Hanzelmann et al., 2013), and GSEAbase (https://bioconductor.org/packages/GSEABase/) package to conduct the entire annotation. Briefly, vt-normalize was used to normalize the variants in the VCF file, then split multi-allele variants into different records. Then, CCAS used Ensembl-VEP to annotate variants with data from the Ensembl database, and used ANNOVAR to annotate the VCF file with dbNSFP, 1000 Genomes Project, ExAC, gnomAD, and dbSNP database. Vcfanno was used to annotate variants with the Cancer Hotspots and Cancer Genome Interpreter database. Output of this part was an annotated VCF file. At the gene level annotation part, annotated VCF and data in other level were converted in to json format. All abnormalities in different level were converted to gene level and were annotated by multiple databases including DGIdb, CancerMine, Reactome, intOGen, Open Target Platform, UniProtKB, and ClinicalTrials.gov. User specified reference genome version was used both in variant level annotation and gene level annotation. At the patient level annotation part, Disease Ontology, ClinicalTrials.gov, and Reactome were used. Briefly, Disease overview information were extracted from Disease Ontology database. Related clinical trials were annotated according to the cancer subtype by using ClinicalTrials.gov database. Pathways information aggregated related pathway of each abnormal gene. Besides, the mutation signature analysis and ssGSEA analysis were performed using DeconstructSigs package, GSVA package, and GSEAbase package (Figure 1). Output of this module had a single sqlite3 database file (https://www.sqlite.org/index.html), an annotated json file, Mutation signature analysis results, and ssGSEA analysis results (Figure 1).

**FIGURE 3:**
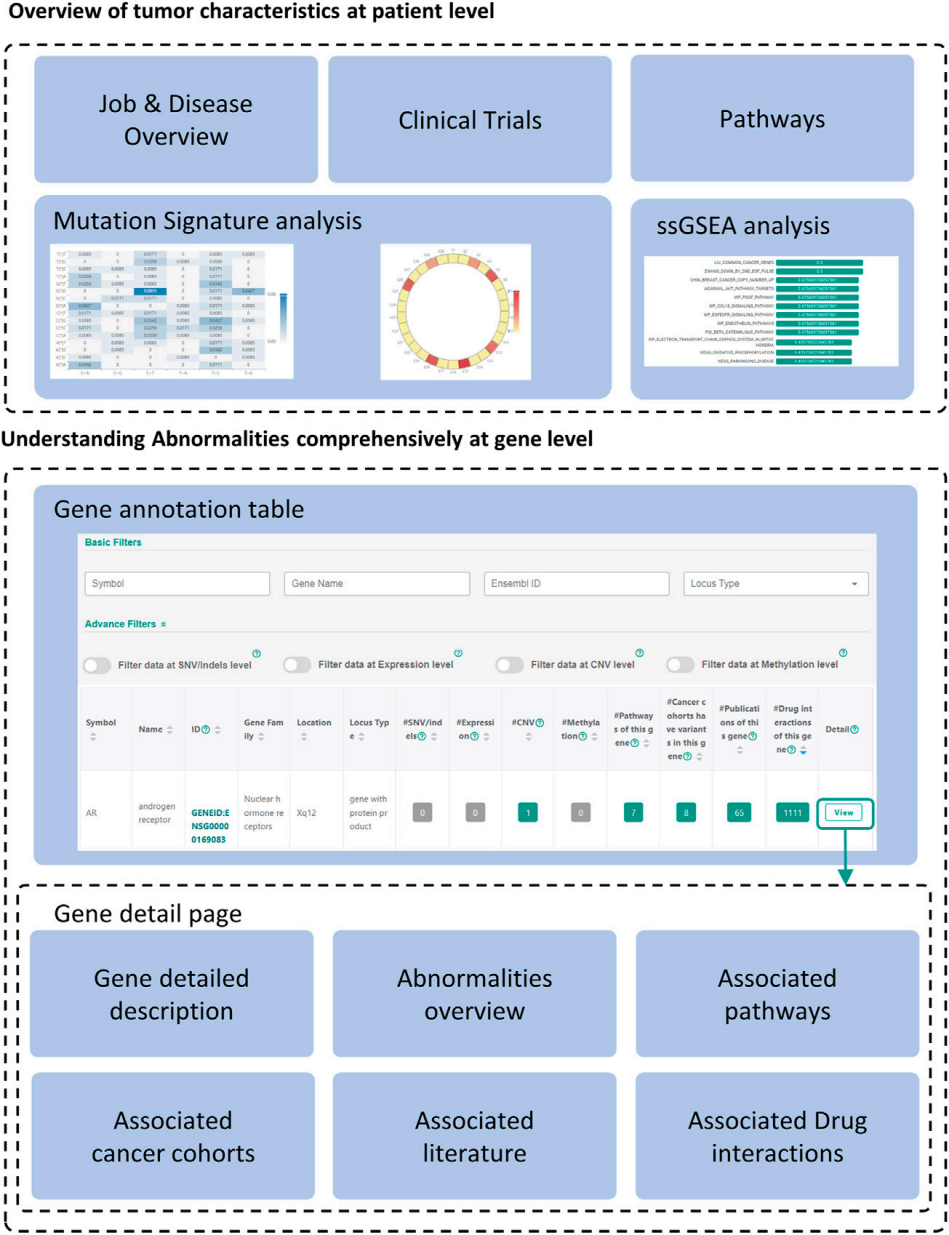
Overview of the annotation results. The annotation results of CCAS consists of patient level annotation and gene level annotation. Gene detailed pages can be viewed by clicking “View” button at the end of each record in the gene annotation table.

The Interpretation module was used to help users understand the annotation results (Figure 1). The annotation results can be divided into two parts: patient level annotation and gene level annotation (Figures 1, 3). The patient level annotation results included Disease overview, Mutation signature analysis, ssGSEA, Clinical trials and Pathways. The gene level annotation results included a gene annotation table and gene detail pages for each gene. Annotation results from variant level databases were integrated in the gene detail page for each gene. Several filters were built in CCAS to help users to filter abnormalities. Basic filters were used to filter abnormalities by gene symbols, gene names, Ensembl gene IDs and locus types. Advance filters were built to data at different level. For SNV/Indels level data, CCAS provided filters based on the IMPACT value from the Ensembl-VEP tool. For other level data, range filters were developed for filtrations (Figure 1; Supplementary Figure S2A).

### 2.5 Web server implementation

The web application was compatible with major web browsers, including Firefox and Chrome. CCAS used the front-end and back-end separation mode. The back-end APIs was built using FastAPI (https://fastapi.tiangolo.com/). MySQL relational database was used for data storage. The front-end pages were constructed using Vue.js (https://vuejs.org/index.html) along with Vue-router (https://router.vuejs.org/) and Vuex (https://vuex.vuejs.org/index.html). Axios (http://www.axios-js.com/) was used to send AJAX requests to convey data from the back-end. The whole system was deployed in the Nginx server (http://nginx.org/). CentOS (https://www.centos.org/) was used to host pipelines and web applications. Back-end job queue and annotation pipeline were built by using Python, R and Shell scripts, and running for each user submission.

### 2.6 Run annotation

Users can submit a job on the home page, fill in the job title and notification email, select the cancer type, upload the file and specify the file type. Submission is started by clicking the “Start” button (Figure 2A). Users can check the progress of the annotation on the Check Results page (Figure 2B). An email notification will be sent to the user at the beginning and the end of the job (Figure 2C). The whole annotation process typically takes around 5–10 min, but depends on the size of the uploaded data.

## 3 Results

### 3.1 Glance of the annotation results

The annotation results of CCAS can be divided into two parts: patient level annotation and gene level annotation (Figure 3). The patient level annotation provides a whole picture on patient’s tumor characteristics. The gene level annotation offers a summarizing table combined with filters and gene detail pages for each abnormal gene (Figure 3). Tables can be searched, sorted, and downloaded. Most figures are interactive to show additional information. In a word, users can easily understand the tumor characteristics and screen for key functional genes in the individual patient level by using CCAS.

#### 3.1.1 Overview of tumor characteristics at patient level

Patient level annotation presents the overview of the patient’s tumor. CCAS shows the type of data submitted by the user, the synonymy of the disease and the associated IDs in the “Job & Disease Overview” section. In order to decipher biological processes involved in tumorigenesis, CCAS calculates the mutation signature based on the patient’s SNV profile and compares it with COSMIC mutation signatures in the “Mutation Signatures” section. In addition, CCAS provides the results of the ssGSEA analysis, which helps user to gain insight into the patient’s tumor characteristics from the enriched gene sets in the “ssGSEA” section. Furthermore, CCAS provides disease-related clinical trials to help users understanding the progress of cancer treatment in the “Clinical trials” section. Finally, CCAS provides the pathways consisting of all the abnormal genes in the “Pathways” section (Figure 3; Supplementary Figure S1).

#### 3.1.2 Understanding abnormalities comprehensively at gene level

The gene annotation table provides a detailed view of abnormal gene functions. The left side of the table shows basic information, including gene symbol and gene name. The right side shows the number of annotation hits in patient’s multi-omics data and the resources integrated in CCAS (Figure 3; Supplementary Figure S2B).

Gene detail pages are used to display comprehensive information about genes. Gene basic information, including gene IDs in various databases, and gene functional description are shown on the top of the page (Figure 3; Supplementary Figure S3A). The lower part of the page shows the patient’s abnormalities in the gene (Figure 3; Supplementary Figures S3B–G). Especially, CCAS describes the abnormalities at the SNV/Indels level, including the frequency of variants in normal and cancer populations, and damage predictions. This helps users to gain deep insight into the variants. In addition, CCAS provides pathway information to help users to understand the gene function (Figure 3; Supplementary Figure S3C). Gene frequencies are provided if the gene has been detected in cancer cohorts (Figure 3; Supplementary Figure S3D). The Literature section provides current research status on this gene (Figure 3, Supplementary Figure S3E). Finally, CCAS offers interactions of genes and drugs, which helps users to evaluate whether a gene is targetable (Figure 3, Supplementary Figures S3F,G).

### 3.2 Identifying key functional genes at individual patient level

Key functional genes not only have significant functional changes in patients’ tumors, but also play a key role in tumorigenesis. Because of the heterogeneity between tumors, these key functional genes may be different at the individual patient level and have not been well studied in the current tumor type. CCAS provides complete annotation on those genes by transferring knowledge across cancer subtypes and databases. Based on the CCAS annotation results, users can find key functional genes through a two-step process. Firstly, filters can be used to screen significant functionally changed genes. The basic filters can filter genes by gene symbols, gene names, Ensembl gene IDs and locus types. Advanced filters can be applied to specific data types (SNV/Indels, expression, CNV and methylation) (Figure 3; Supplementary Figure S2A). Secondly, essential genes in tumorigenesis are screened by examining information in associated literature, drug interactions, pathways, and cancer cohorts (Figure 3).

### 3.3 Case study

To evaluate the performance of CCAS, we carry out a case study for a patient with prostate cancer (DOID:10283). The patient’s multi-omics data are downloaded from GDC data portal (https://portal.gdc.cancer.gov/) at SNV/Indels level (MAF format), expression level (tabular format), CNV level (tabular format) and methylation level (tabular format). We perform basic filtering on data at expression level, CNV level and methylation level to simulate input data by users (Details can be found at https://ngdc.cncb.ac.cn/ccas/docs/#/, 5. Case study). The results of the case study can be viewed by clicking the demo button on the home page or the check results page. At the patient level annotation, the mutation signature analysis reveals that the tumor cells may have a deficiency of DNA mismatch repair function. ssGSEA analysis indicates that multiple cancer related pathways harbor abnormalities including the *AKT* pathway and the *PDGF* pathway, which are consistent with previous studies (van der Poel, 2004; Shorning et al., 2020; Shen et al., 2021). At the gene level annotation, by selecting high impact variant at the SNV/Indels level filter above the gene annotation table, users obtain four genes with significant functional alterations: *ARID1A*, *ZFHX3*, *GADL1*, and *ARID2*. Based on the results, *ARID1A* has 2 related pathways, 70 related cancer cohorts, 55 related publications, and 7 related drug interactions. The gene detail page of *ARID1A* shows that *ARID1A* is a subunit of the SWI/SNF chromatin remodeling complex, and plays an important role in changing chromatin structure by altering DNA-histone contacts within a nucleosome in an ATP-dependent manner. Abnormalities occur at SNV/Indels levels (Abnormalities in user’s upload data section). Moreover, *ARID1A* is involved in 2 pathways including the *RUNX1* pathway, which plays an important role in the development of leukemia (Pathways section) (Kaisrlikova et al., 2022). The literature section indicates that *ARID1A* is observed in a variety of cancers including bladder cancer (Saito et al., 2018; Cao et al., 2020), ovarian cancer (Kim et al., 2016), liver cancer (Sun et al., 2017) and colon cancer (Mathur et al., 2017; Iftekhar et al., 2021). The Cancer cohorts section also reveals *ARID1A* mutations in multiple cancer subtypes, which is consistent with the Literature section. The Drug interactions section suggests that Atezolizumab is likely to interact with this gene. In summary, we suggest that although *ARID1A* is not frequently mutated and well-studied in prostate cancer, it may be one of the important factors in tumorigenesis of prostate tumors and may act as a potential biomarker for this cancer.

Taken together, we conclude that CCAS provides complete annotation on the individual cancer genome both at patient level and gene level by integrating 20 data resources. Especially, genes which are not frequently mutated and well-studied in the patient’s cancer subtype can be well annotated in CCAS.

## 4 Discussion

Cancer is known as a complex disease and is often driven by abnormalities in key cancer genes that occur in cells at multiple omics levels (Chakraborty et al., 2018; ITP-CAoWG, Consortium, 2020). With the explosion of cancer genome data, cancer genome annotation has become an effective way to uncover the underlying mechanisms of tumorigenesis and help the development of treatment strategies (Tebani et al., 2016; Olivier et al., 2019). However, there are still some challenges to be addressed. Firstly, inter-tumor heterogeneity, as a fundamental characteristic of cancer genome, causes incomplete annotation in individual patients. Abnormalities that play crucial roles in individual patients may have low population frequencies and may not be well studied in the cancer type. Secondly, knowledge which is important for cancer genome annotation is usually deposited in different databases with various data structure. Finally, vital knowledge such as clinical trials, drug interactions, literature is lacking in cancer genome annotation.

Existing tools have been developed to facilitate annotation on cancer genome but have limitations on providing more comprehensive annotation for individual patients at multi-omics level. CCAS is designed to annotate multi-omics data from the individual patient and has the following features: Firstly, CCAS has built two ontology frameworks to integrate resources. To date, CCAS has enrolled 20 widely recognized databases in the field. Secondly, within CCAS, knowledge about normal/cancer cohorts, clinical trials, literature, and drug interactions are integrated, providing deep insights into patient’s tumor characteristics. Thirdly, genes which are not frequently mutated and well-studied in one cancer subtype can be well annotated in CCAS by transferring knowledge from other cancer subtypes. This can help users to understand deeply of heterogenous cancer genomes with the aid of existing knowledge across cancer subtypes. Moreover, CCAS provides a two-step process to identify key functional genes that are significantly changed in the patient and play important roles in tumorigenesis, which may provide aid to biomarker identification. Finally, CCAS has a user-friendly web interface, one-click input data submission, smooth and efficient data analysis. No installation or command lines skills are necessary for using CCAS, making it very efficient for users. The current version of CCAS still has some shortcomings, which only integrates knowledge in the resources but with the lack of consensus score to evaluate abnormalities in patients. In a future version, we plan to design an algorithm to support consensus ranking score for each abnormality.

## Data Availability

The original contributions presented in the study are included in the article/Supplementary Material, further inquiries can be directed to the corresponding authors.
