# Supplementary material for: CCAS: One-stop and comprehensive annotation system for individual cancer genome at multi-omics level
Source: Front Genet. 2022 Aug 11;13:956781. doi: 10.3389/fgene.2022.956781 (PMC9403316; doi:10.3389/fgene.2022.956781)
Supplement: Supplementary file 1 [file Presentation1.PPTX]

## Slide 1
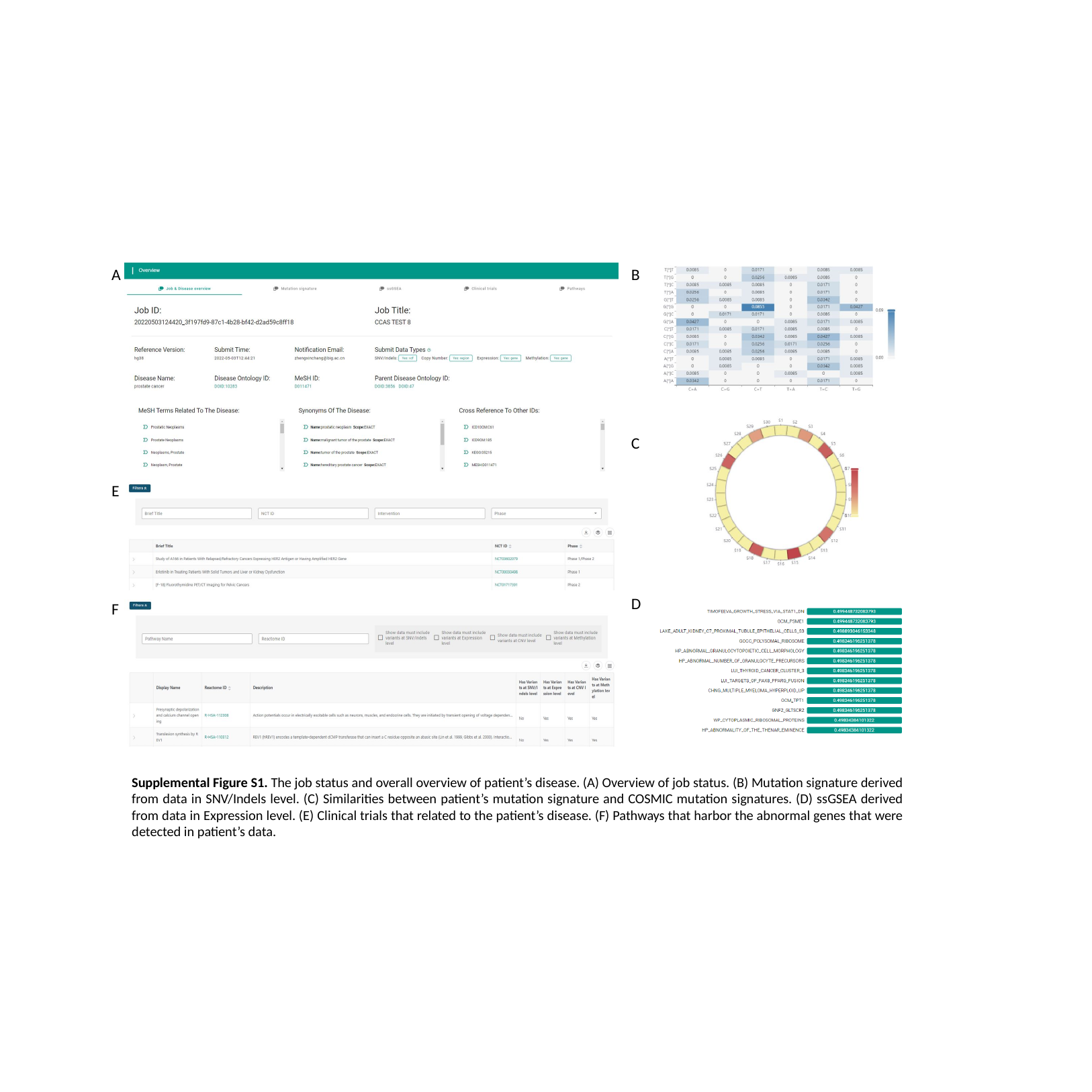

A
B
C
E
D
F
Supplemental Figure S1. The job status and overall overview of patient’s disease. (A) Overview of job status. (B) Mutation signature derived from data in SNV/Indels level. (C) Similarities between patient’s mutation signature and COSMIC mutation signatures. (D) ssGSEA derived from data in Expression level. (E) Clinical trials that related to the patient’s disease. (F) Pathways that harbor the abnormal genes that were detected in patient’s data.

## Slide 2
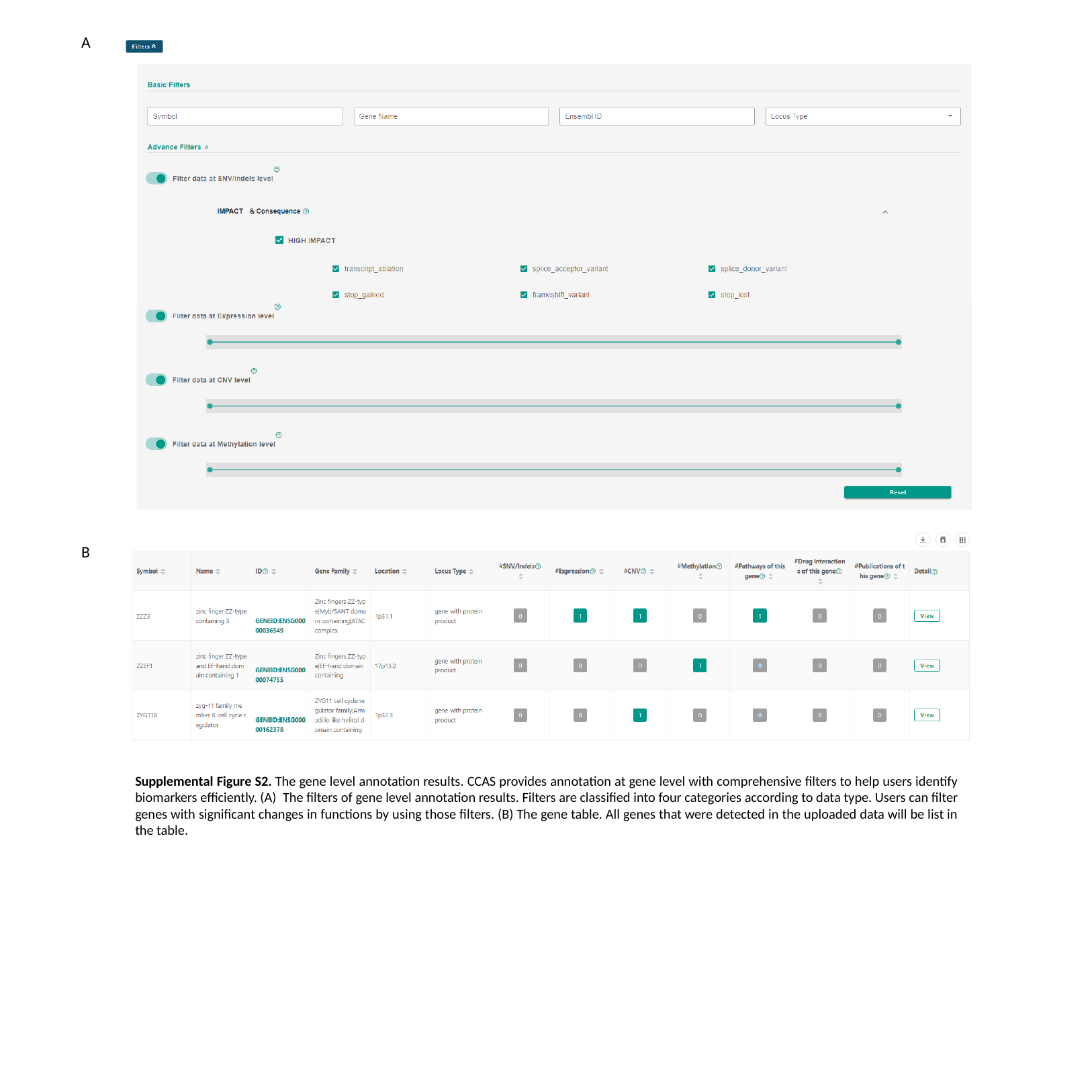

A
B
Supplemental Figure S2. The gene level annotation results. CCAS provides annotation at gene level with comprehensive filters to help users identify biomarkers efficiently. (A) The filters of gene level annotation results. Filters are classified into four categories according to data type. Users can filter genes with significant changes in functions by using those filters. (B) The gene table. All genes that were detected in the uploaded data will be list in the table.

## Slide 3
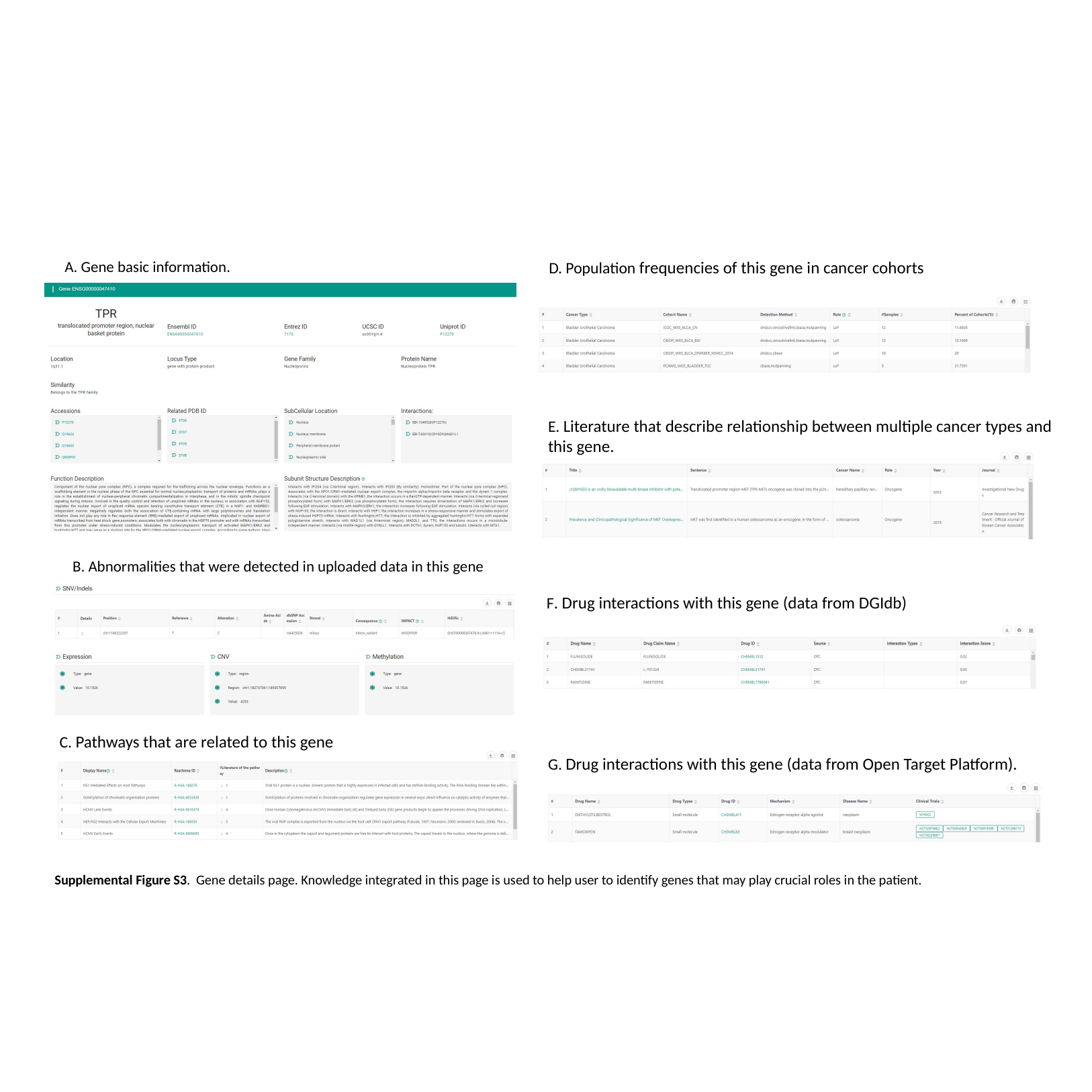

A. Gene basic information.
D. Population frequencies of this gene in cancer cohorts
E. Literature that describe relationship between multiple cancer types and this gene.
B. Abnormalities that were detected in uploaded data in this gene
F. Drug interactions with this gene (data from DGIdb)
C. Pathways that are related to this gene
G. Drug interactions with this gene (data from Open Target Platform).
Supplemental Figure S3. Gene details page. Knowledge integrated in this page is used to help user to identify genes that may play crucial roles in the patient.
